# Fiber Bragg Grating-Based Sensors and Systems

**DOI:** 10.3390/s21248225

**Published:** 2021-12-09

**Authors:** Oleg G. Morozov

**Affiliations:** Department of Radiophotonics and Microwave Technologies, Kazan National Research Technical University Named after A.N. Tupolev-KAI, 10 Karl Marx Str., 420111 Kazan, Russia; OGMorozov@kai.ru or microoil@mail.ru

Today, no one doubts that fiber Bragg gratings (FBGs) have become the most used tool for measuring various physical parameters, the structural integrity of engineering systems, and the biological activity of living systems. Classical approaches to measurements based on temperature and mechanical deformations and changes in the refractive index of the surrounding sensor environment are currently undergoing further active development. New measurement principles are emerging that are based, for example, on physical changes in the length of the grating. The search for ways to simplify and reduce the cost of FBG interrogation systems, on the one hand, and improve their metrological characteristics, on the other, is ongoing. One of the more successful directions of such studies is the transition to microwave photonics measurement systems, which have been developed on the basis of schemes of optoelectronic generators, frequency mapping, probing using multifrequency laser radiation with differences in frequencies lying in the microwave range, and comb generators. A second promising direction is the development and creation of addressable FBGs, the use of which makes it possible to increase the efficiency of processing measurement data and provides the ability to visualize quasi-distributed sensors and map their readings. As for sensor systems based on classical FBGs, it should be said that the resources of new solutions in the design and methodology of interrogators for determining their Bragg wavelengths are far from exhausted.

Presented in this Special Issue is a collection of papers that focus on some of the recent advances related to fiber Bragg grating-based sensors and systems. This Special Issue can be divided into three parts according to convention: intelligent systems, new types of sensors, and original interrogators.

## 1. Intelligent Systems

F. Heilmeier et al., accomplished closing the gap between the external load and internal straining obtained from cast-in FBG, through the development of a fully descriptive FE model considering the contact between casting and glass fiber [1]. This enabled the generation of valuable information about the mechanisms within the strain transition obtained from strain evolution directly around the fiber. The knowledge that FBG functions similarly in internal strain sensors to how it functions in common external strain sensors will allow the application of FBG in actual structural parts and measurements under real load conditions.

S. Gilbertson et al., measured dynamic elastic strain in ~1.8 and 1.0 m diameter containment vessels containing a high-explosive detonation using an array of fiber Bragg gratings [2]. The all-optical method, called real-time localized strain (RTLS) measurement, recorded the strain for 10 ms after detonation with additional measurements being sequentially made at a rate of 1.7 MHz. Using RTLS diagnostics, it was found that the lightweight plastic versions of the FBG strain gauges were significantly more reliable than the heavier metal-backed gauges. The reduced noise present in optical versus electrical strain gauges showed improved accuracy of the instantaneous strain measurement.

D. Cristiani et al., presented a system for rotorcraft landing simulation [3]. The FBG sensors of a system were placed in the most loaded rotorcraft areas, namely the frames and the tail boom; thus, three arrays of strain-measuring sensors were available for a total of 48 sensors, two of which were designed for temperature compensation. The FBG sensors located on the frames measured the strain along the frame circumferential direction; the ones located on the tail boom instead measured the axial strain with respect to the tail boom geometry. FBGs provided key data revealing the structural strains resulting from the landing event loads for the evaluation model of a coupled sequential approach for rotorcraft landing simulation.

Jun Sik Kim et al., designed a wearable hand module which was made of five fiber Bragg grating (FBG) strain sensors and algorithms to achieve high accuracy, even when worn on different hand sizes of users [4]. For real-time calculation with high accuracy, FBG strain sensors moved continuously according to the size of the hand and the bending of the joint. The average error angle of the wearable hand module was observed to be 0.47 ± 2.51° and a mean absolute error (MAE) was achieved at 1.63 ± 1.97°. These results showed that more accurate hand modules than other glove modules applied to different hand sizes can be manufactured using FBG strain sensors that move continuously, with algorithms for tracking these movable FBG sensors.

Jie Wei et al., provided a novel icing-detection technology for composite insulators in transmission lines [5]. As conventional methods have met difficulties in harsh weather, a 110 kV composite insulator with embedded fiber Bragg gratings (FBGs) was proposed for detecting glaze icing in this paper. FBG temperature compensation sensors in ceramic tubes were adopted for simultaneous measurement of icicle loads and temperature. The results show that temperature sensitivities of FBG strain sensors and FBG temperature-compensation sensors were 18.16 and 13.18 pm/°C, respectively. In addition, wavelength shifts were linearly related to icicle loads within the polar angle range of −60° to 60°, and the load coefficient of FBG facing the icicle was −34.6 pm/N.

## 2. New Types of FBG Sensors

Multi-addressed fiber Bragg structures (MAFBS) for microwave-photonic sensor systems were presented by O. Morozov et al., in [6]. The MAFBS is a special type of FBG, the reflection spectrum of which has three (or more) narrow notches. The frequencies of narrow notches are located in the infrared range, while differences between them are located in the microwave range. All cross-differences between optical frequencies of single MAFBS are called the address frequencies set. When the additive optical response from a single MAFBS passed through an optic filter with an oblique amplitude–frequency characteristic is received on a photodetector, the complex microwave signal, which consists of all cross-frequency beatings of all optical frequencies that are included in this optical signal, is taken at its output. This complex microwave signal at the photodetector output contains enough information to determine the central frequency shift of the MAFBS.

T. Agliullin et al., discussed and addressed fiber Bragg structures (AFBS) with two symmetrical phase shifts and their application in load-sensing wheel hub bearings [7]. A prototype load-sensing bearing was instrumented with a single AFBS sensor and mounted in a front right wheel hub of an experimental vehicle. The experimental setup demonstrated comparable results with the simulation during the braking test. The proposed system with load-sensing bearings is aimed at the estimation of loads acting on the wheels, which serve as input parameters for active safety systems, such as automatic braking, adaptive cruise control, or fully automated driving, in order to enhance their effectiveness and vehicle safety.

A. Gizatulin et al., considered the process of fiber vortex mode generation using chiral periodic structures that include both chiral optical fibers and chiral (vortex) fiber Bragg gratings (ChFBGs) [8]. A generalized theoretical model of the ChFBG was developed, including an arbitrary function of apodization and chirping, which provided a way to calculate gratings that generate vortex modes with a given state for the required frequency band and reflection coefficient. In addition, a matrix method was proposed for describing the ChFBG based on the mathematical apparatus of the coupled mode theory and scattering matrices. Simulation modeling of the considered fiber structures was carried out. It was proposed that ChFBGs be used as sensors of physical fields (temperature, strain, etc.), which can be applied to address multisensor monitoring systems due to a unique address parameter—the orbital angular momentum of optical radiation.

Shi-Zhi Chen et al., proposed damage-detection methods based on long-gauge FBG (LG FBG) for highway bridges [9]. Under these circumstances, LG FBG sensors were developed as a novel optical sensor to measure the macrostrain response of the structure. Based on this sensor, many derived damage detection methods were proposed. Therefore, a strict comparative study on three representative methods using LG FBG was carried out. First, theoretical backgrounds and formats of these methods were reformulated and unified for better comparison. Then, based on validated vehicle–bridge coupling simulation, performance of these methods was tested through a series of parametric studies, including various damage scenarios, vehicle types, speeds, road roughness, and noise levels. The precision and reliability of the three methods was thoroughly studied and compared.

## 3. Original Interrogators

In [10], E. Muslimov et al. evaluated the application of curved detectors and freeform optics technologies for the design of FBG interrogation monitors. It was shown that in a high-dispersion spectrograph scheme, the camera part operates in special conditions, which results in a field curvature change. This field curvature can be compensated by the use of a curved detector. When used together with freeform optics, the curved detectors allow for a reduction in the number of optical components to two, or even one element, by merging their functions. Three design examples for the range of 810–860 nm reaching the spectral resolution limit of 89–139 pm at NA = 0.14 are presented, to demonstrate the achieved performance and the technological trade-offs.

In [11], J.B. Rosolem et al., demonstrated a filterless, multipoint, and temperature-independent FBG dynamical demodulator using pulse-width modulation (PWM). In this approach, the FBG interrogation system was composed of a tunable laser and a demodulator that was designed to detect the wavelength shift of the FBG sensor without any optical filter, making it very suitable for use in harsh environments. The authors applied the proposed method, which used the PWM technique for FBG sensors placed in high-pressure and high-temperature environments. The proposed method was characterized in the laboratory using an FBG sensor modulated at a frequency of 6 Hz, with a 1 kHz sweeping frequency in the wavelength range from 1527 to 1534 nm. In addition, the method was evaluated in a field test in an engine of a thermoelectric power plant.

In [12], F. Ouellette showed how dual-wavelength differential detection can be used to measure fiber Bragg grating sensors using nanosecond pulses from a single DFB laser diode by taking advantage of its dynamic chirp. This can be performed in two ways: by measuring the reflected power from two separate pulses driven by two different currents, or by taking two delayed digitized samples within a single pulse. A prototype instrument using fast digitizing and processing with an FPGA was used to characterize the chirp, from which the performance can be optimized for both measurement schemes.

This Special Issue *Fiber Bragg Grating-Based Sensors and Systems* presents a collection of cyber-physical tasks that are far from completely solved. We hope this Special Issue will serve as a reference to audiences who are interested in challenging cyber-physical problems in the industry and in science applications. We would be very pleased if researchers or engineers from academic or industrial backgrounds were inspired by the content presented in this Special Issue.
